# A Soft-Reference Breast Ultrasound Image Quality Assessment Method That Considers the Local Lesion Area

**DOI:** 10.3390/bioengineering10080940

**Published:** 2023-08-07

**Authors:** Ziwen Wang, Yuxin Song, Baoliang Zhao, Zhaoming Zhong, Liang Yao, Faqin Lv, Bing Li, Ying Hu

**Affiliations:** 1 School of Mechanical Engineering and Automation, Harbin Institute of Technology, Shenzhen 518055, China; wangziwen@stu.hit.edu.cn; 2 Shenzhen Institute of Advanced Technology, Chinese Academy of Sciences, Shenzhen 518055, China; yx.song@siat.ac.cn (Y.S.); liang.yao@connect.um.edu.mo (L.Y.); ying.hu@siat.ac.cn (Y.H.); 3 The Second School of Clinical Medicine, Southern Medical University, Guangzhou 510515, Chinalvjin8912@163.com (F.L.); 4 Department of Ultrasound, The Third Medical Centre of Chinese PLA General Hospital, Beijing 100039, China

**Keywords:** breast ultrasound, image quality assessment, soft reference

## Abstract

The quality of breast ultrasound images has a significant impact on the accuracy of disease diagnosis. Existing image quality assessment (IQA) methods usually use pixel-level feature statistical methods or end-to-end deep learning methods, which focus on the global image quality but ignore the image quality of the lesion region. However, in clinical practice, doctors’ evaluation of ultrasound image quality relies more on the local area of the lesion, which determines the diagnostic value of ultrasound images. In this study, a global–local integrated IQA framework for breast ultrasound images was proposed to learn doctors’ clinical evaluation standards. In this study, 1285 breast ultrasound images were collected and scored by experienced doctors. After being classified as either images with lesions or images without lesions, they were evaluated using soft-reference IQA or bilinear CNN IQA, respectively. Experiments showed that for ultrasound images with lesions, our proposed soft-reference IQA achieved PLCC 0.8418 with doctors’ annotation, while the existing end-to-end deep learning method that did not consider the local lesion features only achieved PLCC 0.6606. Due to the accuracy improvement for the images with lesions, our proposed global–local integrated IQA framework had better performance in the IQA task than the existing end-to-end deep learning method, with PLCC improving from 0.8306 to 0.8851.

## 1. Introduction

Breast disease is the most serious problem for women’s health today and has become a serious global public health problem [1]. Studies have shown the importance of early screening for the prognosis and treatment of breast cancer [2]. Ultrasound (US) examination is widely used for the diagnosis of internal organs and superficial organs due to its non-invasiveness, non-radiation use and convenience, compared with CT, MRI or X-ray [3]. In particular, it has been proved to be especially effective in the diagnosis of breast cancer [4]. Meanwhile, ultrasound examinations rely on clinical experience, which affects the quality of ultrasound images and the accuracy of diagnosis. Therefore, objective quality evaluation of ultrasound images has clinical significance to guarantee the standardization of ultrasound examination. In addition, to achieve automation of ultrasound examination, the ultrasound scanning robot has emerged as a research trend. In our previous research, an autonomous ultrasound scanning robot was designed [5]. The quality assessment of ultrasound images is considered as a visual servo that can be used as control feedback for autonomous scanning robots. There are many reasons for the poor quality of ultrasound images, such as noise in signal transmission, artifacts, low echoes due to poor contact between the probe and the skin, and dragging blurring due to probe movement. To address these issues, this study focuses on the quality assessment of breast ultrasound images.

Image quality assessment has been intensively studied in the field of nature images. Generally, IQA is divided into subjective assessment, which is judged by experts, and objective assessment, which is computed with mathematical algorithms. Subjective IQA can be time-consuming, labor-intensive, costly, error-prone, and inconsistent [6]. The objective assessment can be further divided into three categories based on the availability of reference images: (i) full reference (FR-IQA), where there is a perfect reference image for comparison with the test image; (ii) reduced reference (RR-IQA), which contains partial information of the reference image, and (iii) no reference (NR-IQA), where no information regarding the reference image is available for the assessment [7]. FR-IQA calculates the statistical difference between the sample image and the reference image, such as PSNR [8], RMSE [9], intensity [10] or SSIM [11]. However, FR-IQA and RR-IQA have limited practical applications since the reference image is usually not available, especially for medical images. In contrast, NR-IQA methods do not require any information from a reference image and are well-suited for practical applications.

Many researchers have focused on the quality assessment of medical images, including MRI [6,12,13], retinal fundus images [14], video endoscopy [15,16], CT [17], and ultrasound images. Medical ultrasound images are known to present with a lot of noise and artifacts, low resolution, blurred boundaries, and low contrast, which bring many difficulties to the quality assessment of images. Traditional NF-IQA methods for ultrasound images generally extract image features first and then adopt SVM, SVR, and other methods with machine learning for quality regression or classification [13]. In recent years, some researchers have introduced confidence mapping into autonomous robotic scanning tasks to evaluate the quality of ultrasound images. Confidence mapping is a method to quantitatively evaluate the uncertainty caused by the attenuation and shadow in ultrasound images [18,19]. However, the confidence map only evaluates the contact condition between the probe and human skin, indicating the reliability of the ultrasound imaging at each pixel, and does not consider the significance of the image content for diagnosis. Therefore, the confidence mapping method has a big gap between it and the clinical standard of ultrasound image quality assessment. Recently, end-to-end deep learning methods have been demonstrated to have great potential for IQA. Most researchers working on clinical ultrasound image analysis have proposed to assess the image quality based on whether key anatomical structures can be detected. Wu et al. [20] proposed a computer-assisted fetal ultrasound image quality assessment algorithm, which is composed of L-CNN and C-CNN. The function of L-CNN is to find the ROI (Region of Interest) in the fetal abdominal region of the ultrasound image, and C-CNN detects whether there are key structures, such as gastric vesicles and umbilical veins, in the obtained ROI to evaluate the quality of the ultrasound image. Lin et al. [21] proposed a MF R-CNN (Multi-task Fast Regional Convolutional Neural Network) for the quality assessments of fetal head ultrasound images, which can detect and identify the key anatomical structures of the fetal head, and then evaluate the quality of ultrasound images. Camps et al. [22] proposed a DenseNet classification to automatically assess the quality of trans-perineal ultrasound images of the male pelvic region based on the visibility of the prostate. Similarly, Antico et al. [23] trained a DenseNet to classify knee ultrasound images into two categories based on the visibility of cartilage. In breast ultrasound images, the anatomical structures such as the subcutaneous fat layer, breast glands, pleura and other anatomical structures are not clear, and the breast morphology of different patients is very different due to individuality. Therefore, it is difficult to assess the quality of breast ultrasound images through the extraction and detection of specific anatomical structures.

There are some other studies which have converted quality assessments to classification or regression by using doctors’ subjective evaluations of samples as labels. In Zhang’s work, ultrasound images were artificially added with noise, subjectively scored, and regressed with DCNN [24]. In our previous work, doctors classified breast ultrasound images into five levels according to subjective evaluation, and a BCNN assessment network was trained [25]. These works conducted global quality assessment of ultrasound images; however, for medical ultrasound images, doctors usually pay more attention to image content, especially those images with lesions. This research is an extension of our previous work [25], and a soft-reference-based global–local hybrid breast ultrasound IQA framework is proposed. The breast ultrasound images were first classified into two groups that were either with or without lesions, then for the ultrasound images without lesions, the global image quality was calculated, and for the ultrasound images with lesions, the lesion was segmented and the image quality of the local region was evaluated.

The main contributions of this paper are listed as follows:

- An integrated global–local breast ultrasound IQA framework is established, in which the images without lesions are evaluated globally, and the images with lesions are evaluated locally (i.e., with a focus on the lesion area).
- A soft-reference IQA method based on lesion segmentation is proposed, in which the segmented lesion is taken as a reference image and transforms the medical ultrasound image assessment problem, from no reference to reduced reference.
- A comprehensive evaluation index for medical ultrasound images with lesions is proposed, including pixel-level features MSE (Mean Squared Error), PSNR (Peak Signal-to-Noise Ratio) and semantic-level features CR (Contrast Ratio) and SSIM (Structural Similarity).

The rest of this paper is organized as follows. Section 2 describes the dataset construction, which includes the image collection and data labeling and the IQA framework. Section 3 presents the implement details of the network and the results of the experiments. Finally, the conclusion of this study is presented in Section 4.

## 2. Materials and Methods

### 2.1. Data Collection

The ultrasound images used in this paper were collected by five doctors from the ultrasound department of PLA General Hospital, including ultrasound images with and without lesions. The lesions include benign cancers, nodules or cysts (fibroadenoma or fibrocystic breast disease). All the doctors participating in this study had more than five years of clinical experience. The collection period ranged from August 2020 to April 2021. The ultrasound examination equipment used was Siemens S2000, and the probe used was 9L4. Starting with the default parameter setting for breast examination, different doctors adjusted these parameters according to their habits and collected breast images. The collected images were captured by an image acquisition card and saved in PNG format. The images of healthy breasts and images with lesions were used in this research, and the images with missing breasts after mastectomy were excluded. Finally, there was a total of 1285 ultrasound images collected from 524 female patients, including 844 healthy breast ultrasound images (A) and 441 (B) breast ultrasound images with lesions. The age of patients ranged from 18 to 86 years, with an average of 49.5 years. The data collection was approved by the Institutional Review Board under YSB-2020-Y0902. The datasets A and B were divided randomly into the training set (A.1 and B.1), validation set (A.2 and B.2), and test set (A.3 and B.3) according to the ratio of 6:2:2, respectively.

### 2.2. Data Labeling

To improve the credibility of the data labeling, three other doctors with more than ten years of ultrasound examination experience were invited to conduct the image quality assessment. The score ranged from 1 to 4, with 4 indicating excellent image quality that can be used for diagnosis, with 3 indicating defective image quality but which does not affect the diagnosis, with 2 indicating poor image quality which affects the diagnosis, and with 1 indicating unacceptable image quality and cannot be used for diagnosis. The scoring is based on doctors’ clinical evaluation standards including the amount of noise, the definition, the contrast, and the vision field of the images. The final score of each image was the averaged value from different doctors and rounded to an integer. The numbers of breast ultrasound images with their different quality scores are shown in Table 1. Additionally, the 441 breast ultrasound images with lesions were manually segmented.

### 2.3. Proposed Framework for Breast Ultrasound Image Quality Assessment

**Figure 1:**
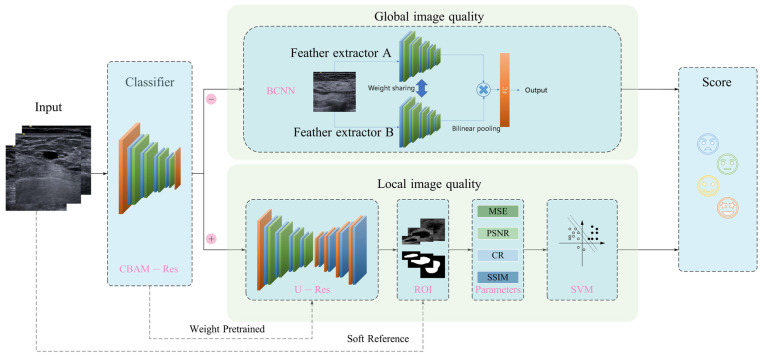
Global–local integrated breast ultrasound IQA framework.

Most of the existing breast ultrasound IQA methods focus on the global image quality, but ignore the image quality of the lesion area, which determines the diagnostic value of ultrasound images. Hence, in this section, a global–local integrated quality assessment framework for breast ultrasound images is presented. Figure 1 provides an overview of the process. The proposed IQA system has three major modules: firstly, a binary classification of breast ultrasound images is implemented to determine whether the input is negative (without lesions) or positive (with lesions), using a residual network containing an attention module. Secondly, for breast ultrasound images without lesions, a global image quality evaluation network based on BCNN [25] is used. Thirdly, for breast ultrasound images with lesions, a local IQA method based on breast lesion segmentation is proposed. For the local image quality assessment, the result of lesion segmentation is used as a mask to compare with the local lesion area of the breast ultrasound image, then IQA metrics are calculated to measure the pixel-level differences and structural differences between them; finally, these metrics are inputted to an SVM (Support Vector Machine) to obtain the image quality of the local lesion area. By taking the segmented lesion as a reference, the traditional medical ultrasound image assessment problem is transformed from no reference to reduced reference, so we define this method as soft reference (SR) in this research. Meanwhile, positive samples and negative samples were evaluated using different models to improve the diagnosability of ultrasound images in clinical applications.

### 2.4. Classification Network

**Figure 2:**
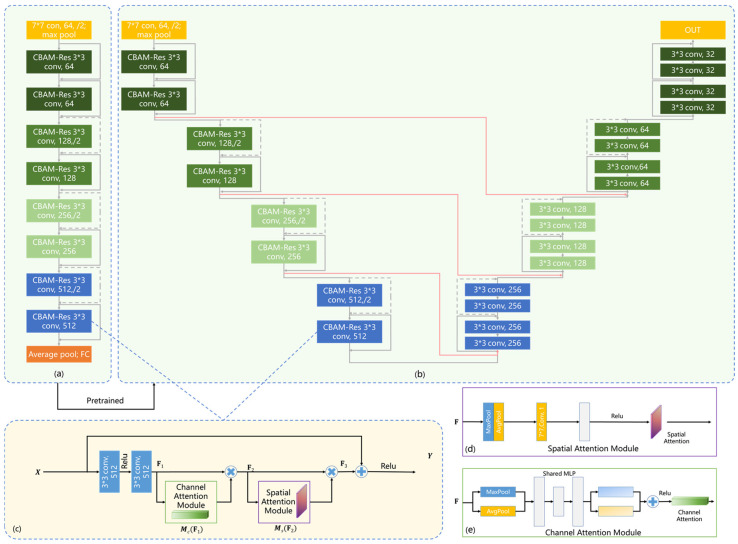
Classification and segmentation networks with shared weights. (**a**) CBAM-ResNet18 classification network. (**b**) U-Res-CBAM segmentation network. (**c**) Residual block with CBAM module. (**d**) Spatial attention module. (**e**) Channel attention module.

ResNet is one of the most commonly utilized networks in image classification, whose main contribution is the residual learning module, which solves the network degradation problem caused by the gradient disappearance of CNN [26]. Considering the real-time performance of the network and the size of the existing training data, ResNet18 was selected as the basic component of the feature extractor in this research. In addition, the lesion identification of breast ultrasound images mainly relies on local features. Hence, to further improve the model performance, we introduce the convolutional block attention module (CBAM) [27] into our network to select the features containing more information and suppress less useful features. In this research, the breast lesion detection network (CBAM-ResNet18) contains 8 CBAM-Res modules inserted into the Residual Block, as shown in Figure 2a,c. CBAM includes a spatial attention module and a channel attention module. The spatial attention module can find out the areas that need to be paid attention to in the image. As shown in Figure 2, global average pooling (GAP) and global maximum pooling (GMP) are performed along the channel direction for the input feature matrix $F\in {\mathbb{R}}^{H*W*C}$, followed by a 7 × 7 convolution and activation function. The spatial attention vector can be expressed as Equation (1).

(1) $$M_{s}\left(F\right)=\sigma \left\{f^{7\times 7}\left[AvgPool\left(F\right);MaxPool\left(F\right)\right]\right\}$$

where σ represents the activation function ReLu. The channel attention module can find out the features that need to be paid attention to in the image. GAP and GMP are performed by each feature plane, followed by a shared multilayer perception machine. The channel attention vector can be expressed as Equation (2).

(2) $$M_{c}\left(F\right)=\sigma \left\{MLP\left[AvgPool\left(F\right)\right]+MLP\left[MaxPool\left(F\right)\right]\right\}$$

### 2.5. BCNN Global IQA Network

The BCNN architecture has good performance in fine grained image classification task and is introduced in this work to improve the accuracy of breast ultrasound image quality assessment. The application scenario of fine-grained classification network is to distinguish objects from different subclasses within a general category such as identifying the species of a bird or the model of an aircraft. It is with large intra-class variance and small inter-class variance affected by the pose, background, and viewpoint [28]. These characteristics are similar to the quality assessment of medical ultrasound images. The difference between ultrasound images of different qualities is not significant, while ultrasound images of the same image quality may have great differences due to the location and angle of image acquisition. The BCNN architecture uses a quadratic linear pooling operation to combine the features extracted by the feature extractor A and feature extractor B, which comprise ResNet18, as shown in Figure 1. The quadratic linear pooling operation involves carrying out the outer product operation of the features extracted by the two networks. To reduce the number of parameters, we make feature extractor A and feature extractor B share parameters. Only one network model needs to be trained, which shortens the training time.

### 2.6. Lesion Segmentation Network

U-Net is the most successful network structure in the field of medical image segmentation [29]. It uses the classical encoder-decoder structure, consisting of compressed and extended paths. Because the lesion detection task in 2.4 has similar feature extraction process with the lesion segmentation task in this section, the encoder of U-Net is replaced by CBAM-ResNet18 to extract more effective features. The decoder of U-Net is replaced by the basic structure of ResNet18. The encoder uses the lesion detection network in 2.4 as the pre-trained model, and the decoder uses the model pre-trained on ImageNet. The modified U-Net network is named as U-Res-CBAM in this study, as shown in Figure 2b.

### 2.7. Parameters of Local Image Quality Assessment 

**Figure 3:**
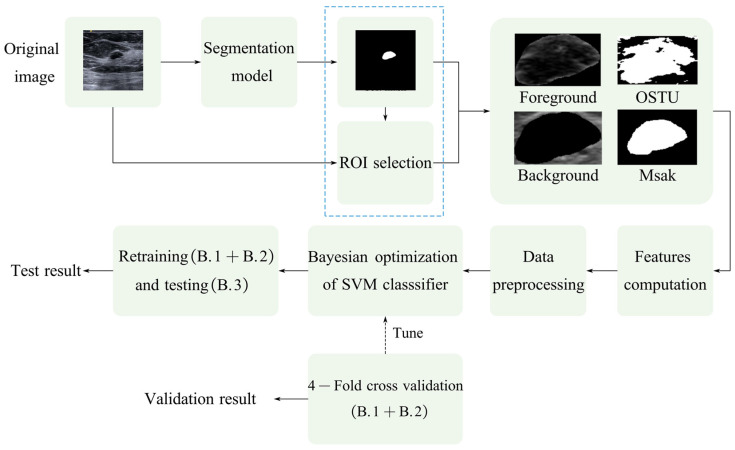
The proposed soft-reference IQA method based on lesion segmentation and SVM classifier.

As shown in Figure 3, for ultrasound images with lesions, we first segment the lesions using a deep learning-based method and an image processing-based method separately. If the local quality of the image is good, then the masks obtained by the two methods should be closer. The local quality can be reflected by calculating the difference between the masks obtained by the two methods. In this way, we convert the no-reference method into a soft-reference method. In this subsection, four parameters are adopted to calculate the image quality of the local lesion region in breast ultrasound images, at both the pixel level and the semantic level.

#### 2.7.1. Mean Squared Error (MSE)

MSE is a common metric used in image processing to evaluate the mean value of the difference between two images, which can be expressed by Equation (3).

(3) $$\mathrm{MSE}=\frac{1}{MN}\sum_{x=0}^{M-1}\sum_{y=0}^{N-1}{\left[A(x,y)-B(x,y)\right]}^{2}$$

where *A*(*x*, *y*) and *B*(*x*, *y*) denote the grayscale values of pixels (*x*, *y*) in image *A* and image *B*, respectively. *M* and *N* denote the number of pixels in the length direction and width direction, respectively.

#### 2.7.2. Peak Signal-to-Noise Ratio (PSNR)

PSNR measures the ratio of the intensity of the peak signal to the average intensity of the noise, and it is changed to decibels (dB) by taking the logarithm of the value, as expressed by Equation (4).

(4) $$\mathrm{PSNR}=10log_{10}\frac{MaxValue^{2}}{MSE}$$

where *MaxValue* indicates the maximum grayscale value.

#### 2.7.3. Contrast Ratio (CR)

CR indicates the degree of distinguishability between the lesion region and the surrounding region of the image. The lesion region in the breast ultrasound image is usually darker than the surrounding region, since the echo of the lesion area is weaker than that of healthy tissue area, presenting a low echo state. The greater the difference in image gray value between the lesion area and the surrounding area, the easier the lesion region is to distinguish, which indicates that the image quality is high. The breast ultrasound image is firstly segmented to obtain its foreground (the lesion region) and background (the surrounding region), and the parameter CR is calculated by Equation (5).

(5) $$\mathrm{CR}=\frac{\left(MeanF-MeanB\right)}{255}$$

where *MeanF* and *MeanB* represent the average grayscale value of the foreground and background, respectively.

#### 2.7.4. Structural Similarity (SSIM)

**Figure 4:**
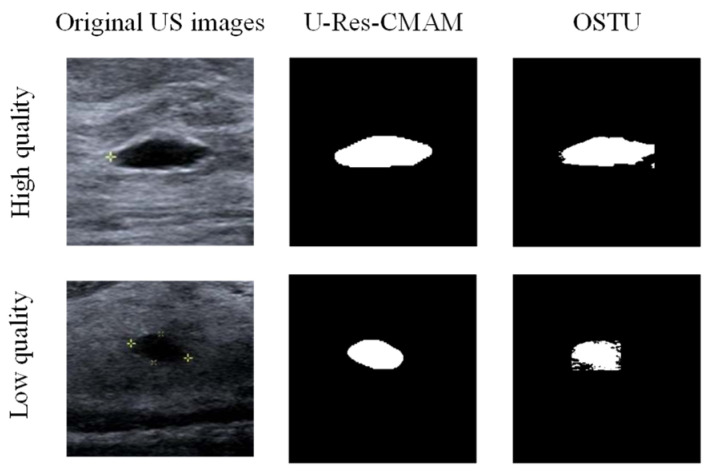
The original ultrasound image, the segmentation mask obtained by the neural network, and the segmentation result obtained by the OSTU algorithm of lesion ROI.

SSIM is adopted to measure the similarity of structure between the lesion region and the reference image mask. In this study, SSIM is defined as the Dice between the segmentation results obtained using the OTSU threshold segmentation algorithm [30] and the reference image mask obtained with the segmentation network described in Section 2.6. Figure 4 shows the lesion segmentation results of high-quality and low-quality breast ultrasound images with the OTSU algorithm and our segmentation network U-Res-CBAM. It is observed that the OTSU algorithm has better segmentation result for a high-quality image, which is with a clear edge, a complete boundary and an obvious difference between the lesion region and the surrounding region. For a low-quality image with a blurred edge, the segmentation result of the OTSU algorithm is poor. Therefore, the closer the segmentation results between those obtained using OSTU algorithm and the reference image mask obtained with the U-Res-CBAM network, the higher the image quality. The parameter SSIM can be calculated by Equation (6).

(6) $$\mathrm{Dice}=\frac{2\left|A\cap B\right|}{\left|A\right|+\left|B\right|}$$

where *A* and *B* represent binary segmented images using the OSTU algorithm and the U-Res-CBAM network.

### 2.8. Support Vector Machine (SVM)

To create a quality model based on the above pixel-level features and semantic-level features, an optimizable SVM classifier is used. Firstly, these features are normalized into [0, 1]. Then the SVM maps the feature vector ***F***_i_ = {*f*_1_, *f*_2_, *f*_3_, *f*_4_} of the images into a subjective score classes space {1, 2, 3, 4}. With training dataset {(***F***_1_, *q*_1_), (***F***_2_, *q*_2_), …, (***F***_N_, *q*_N_)}, the SVM classifier tries to find a hyperplane function that separates classes based on these features, as expressed by Equation (7).

(7) $$f=\omega^{T}\phi \left(F\right)+b=\sum_{i=1}^{N}\alpha_{i}q_{i}\kappa \left(F,F_{i}\right)+b$$

where ***ω*** is the weight vector, *b* is a bias parameter, $\alpha_{i}$ is the combination coefficient, $\kappa \left(F,F_{i}\right)=\exp\left(-\gamma \|F_{i}-F{\|}^{2}\right)$ is a Gaussian kernel function, and *γ* denotes a precision parameter. The determination of *ω* and *b* can be modeled as an optimization problem, as expressed by Equation (8).

(8) $$\begin{array}{c} \underset{\omega ,b,\xi_{i}}{\min}\frac{1}{2}\|\omega {\|}^{2}+C\sum_{i=1}^{N}\xi_{i}\\ s.t.\{\begin{array}{c} q_{i}\left(\omega^{T}\phi \left(F\right)+b\right)\geq 1-\xi_{i}\\ \xi_{i}\geq 0,i=1,2,\cdots ,N \end{array} \end{array}$$

where $\xi_{i}$ is the slack variable and *C* is a penalty parameter. In this study, the parameters *C* and *γ* of the SVM model are determined using a Bayesian optimization search method [31], tuned by four-fold cross-validation, as shown in Figure 3. Once the hyperparameters are determined, the SVM model is then retrained with the whole training data of breast ultrasound images with lesions. Then, for each testing image, the SVM-based model is used for quality prediction.

## 3. Results

### 3.1. Implementation Details

In this section, we conduct experiments to evaluate the efficacy of the proposed US image quality assessment framework, including classification, global IQA, segmentation, and local IQA. Finally, the proposed approach is compared with our previous US image quality assessment methods. In this study, we used the open-source framework Pytorch for network implementation. The version of Pytorch was 1.7 and the version of Python was 3.7. All the experiments were performed on a workstation with NVIDIA GTX3090 (Intel i7-9700K, 32 G RAM, Windows 10).

### 3.2. Quantitative Evaluation Metrics

In order to quantitatively measure the correlation between the output results of quality assessment network and doctors’ ground-truth manual annotations, four quantitative evaluation metrics are used as the evaluation metrics, as shown in Section 3.2.1, Section 3.2.2, Section 3.2.3 and Section 3.2.4.

#### 3.2.1. Pearson Linear Correlation Coefficient (PLCC)

PLCC is adopted to evaluate the accuracy of the IQA model [7], which can be calculated with Equation (9).

(9) $$\mathrm{PLCC}=\frac{\sum_{i=1}^{N}\left(X_{i}-\bar{X}\right)\left(Y_{i}-\bar{Y}\right)}{\sqrt{\sum_{i=1}^{N}{\left(X_{i}-\bar{X}\right)}^{2}\sum_{i=1}^{N}{\left(Y_{i}-\bar{Y}\right)}^{2}}}$$

where $X_{i}$ and $Y_{i}$ represent the score of doctors’ manual annotation and the score output by the network for the *i-th* image, *N* represents the number of images included in the comparison, and $\bar{X}$ and $\bar{Y}$ represent the averaged values for these images. The value of PLCC ranges from −1 to 1. If the absolute value of the PLCC value is close to 1, it indicates that the output results of quality assessment network and doctors’ ground-truth manual annotations have strong correlation.

#### 3.2.2. Spearman Rank-Order Correlation Coefficient (SRCC)

SRCC is adopted to evaluate the monotonicity of the IQA model [7], which can be calculated with Equation (10).

(10) $$\mathrm{SRCC}=1-\frac{6\sum_{i=1}^{N}d_{i}^{2}}{N\left(N^{2}-1\right)}$$

where *d*_i_ denotes the difference between the subjective quality score ranking and the objective quality score ranking of the *i-th* image. The value of SRCC ranges from 0 to 1. If the absolute value of SRCC is close to 1, it indicates that the two sets of data are completely consistent.

#### 3.2.3. Accuracy

Accuracy indicates the percentage of samples with completely correct predictions.

#### 3.2.4. Root Mean Square Error (RMSE)

RMSE is used to evaluate the consistency of the IQA model with the labels, as shown in Equation (11). *s_i_* and *p_i_* denote the subjective quality score and objective quality score of the *i-th* image, respectively.

(11) $$RMSE=\sqrt{\frac{1}{n}\sum_{i=1}^{N}{\left(s_{i}-p_{i}\right)}^{2}}$$

### 3.3. Classification Experiment

**Figure 5:**
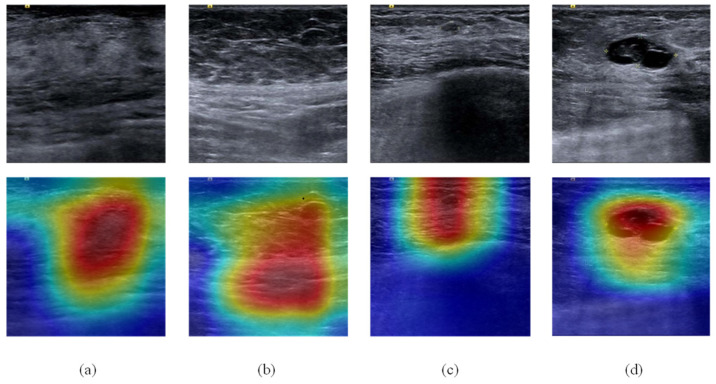
Heat map of the classification network for different ultrasound images. (**a**,**b**) are images without lesion, (**c**,**d**) are images with lesion.

In this sub-experiment, 1285 breast ultrasound images (dataset A and B) were used. The model was trained for 150 epochs with a batch size of 16. The Adam optimizer was adopted with an initial learning rate of 1 × 10^−4^. CrossEntropyLoss was chosen as the loss function, and the classification results were evaluated in term of accuracy. The performance of the model was tested on the validation set (A.2 and B.2) every four epochs, and the model with the best validation accuracy was retained and evaluated on the test set (A.3 and B.3). ResNet18 was initialized using a pre-trained model on ImageNet. To show the effectiveness of our proposed method, three groups of classification experiments were conducted. The first experiment was with the classification model ResNet18 without pre-training, the second experiment was with the classification model ResNet18 with pre-training, and the third experiment was with our proposed classification model CBAM-ResNet18 with pre-training. As shown in Table 2, the classification accuracy of our proposed model was 99.2% on the test set, which is the best among the three methods. Figure 5 shows the heat map of the classification network computed by Grad-CAM [32]. For ultrasound images with lesions, the neural network tends to focus on the lesion, while for images without lesions, the neural network does not have a clear focus.

### 3.4. Global IQA Experiment

For breast ultrasound images without lesions (dataset A), the global IQA network described in Section 3.3 was used. The model was trained for 30 epochs with a batch size of 24. The Adam optimizer was adopted with an initial learning rate of 1 × 10^−4^. MSE was chosen as the loss function. We used ResNet18 pre-trained in ImageNet as the initial state of the feature extractor. The validation set was used to optimize hyperparameters and retain the best model on a certain epoch. Table 3 shows that the global IQA method achieves good correlation with the doctor’s subjective evaluation for images without lesion.

### 3.5. Segmentation Experiment

**Figure 6:**
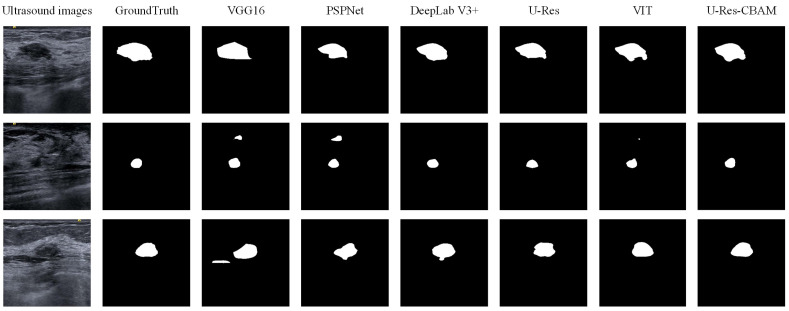
The lesion segmentation results with different networks.

In this subsection, we briefly describe the experiment of breast lesion segmentation, which is a necessary step for local IQA. A total of 441 breast ultrasound images with lesions (dataset B) were used. The model was trained for 100 epochs with a batch size of 16. The Adam optimizer was adopted with an initial learning rate of 1 × 10^−4^. Dice was chosen as the loss function. To show the effectiveness of our proposed segmentation network, contrast experiments were conducted to compare with other state-of-the-art segmentation models including VGG16 [32], PSPNet [33], DeepLab V3+ [34], U-Res [35], and ViT [36]. The initial weights of the encoder used the weights of the classification network in Section 3.3. As shown in Table 4, the U-Res-CBAM network achieved the best segmentation results, with Dice 0.9272 on the validation set and Dice 0.9126 on the test set. Figure 6 shows the lesion segmentation results of the ultrasound images with different segmentation networks.

### 3.6. Local IQA Experiment

**Figure 7:**
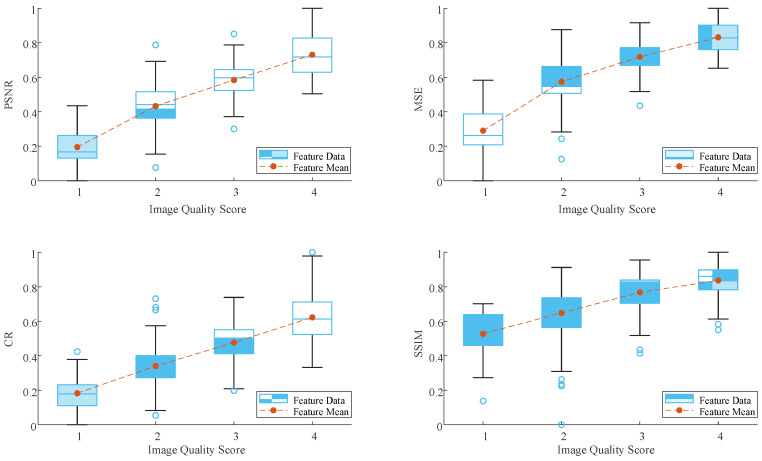
Data distribution of the four parameters with respect to different quality scores.

For 441 breast ultrasound images with lesions (dataset B), the local soft-reference image quality assessment (SF-IQA) method was adopted. Firstly, four local IQA parameters were calculated. Then the values were normalized to [0, 1]. We took 1 minus the normalized result of MSE, so that all the four quality evaluation parameters were positively correlated with the quality degree of the images. A box-plot of the four parameters with respect to the different quality scores is shown in Figure 7. It is noted that the mean value of each parameter increases monotonically with image quality, which confirms the rationality and feasibility of adopting these four parameters to quantify the image quality. A fourfold cross-validation method was used to find the best SVM parameters and the test set was used to evaluate the performance of the local SF-IQA model. The results using the mask obtained with different lesion segmentation methods as reference images are shown in Table 5. For the U-Res-CBAM segmentation method used in this paper, the best performance was achieved on the test set, with PLCC 0.8418, SRCC 0.8462, Accuracy 0.8068, and RMSE 0.4395. Furthermore, the PLCC between the SVM IQA model and the doctor’s subjective quality evaluation can reach 0.9255 when the reference images of the four quality evaluation metrics are replaced with Ground-Truth manually segmented by the doctor. It is obvious that lesion segmentation accuracy has an important impact on our IQA method. The higher the segmentation accuracy, the better the consistency between the soft-reference IQA and the subjective evaluation of the physician.

### 3.7. Global–Local Integrated IQA Experiment

To evaluate the proposed global–local integrated IQA framework, a contrast experiment was conducted to compare with our previous work which applied only a global BCNN network to assess ultrasound images with and without lesions. For our proposed global–local integrated IQA framework, the global component and local component were trained with dataset A.1 and dataset B.1 separately, and the best hyperparameters were determined with validation datasets A.2 and B.2 separately, which were introduced in Section 3.4. and Section 3.6. Then the test images (A.3 + B.3) were classified and assessed. Meanwhile, for the global BCNN network without consideration of lesions [25], training took place with mixed dataset A.1 + B.1, and the best hyperparameters were determined with mixed dataset A.2 + B.2; the 257 test images (A.3 + B.3) were then assessed.

The experimental results are listed in Table 6. It can be noted that, for the test set consisting of images with lesions (B.3), our proposed SR-IQA achieved PLCC 0.8418 with doctors’ labelling, while the global BCNN network that did not consider the local lesion features only achieved PLCC 0.6606, proving the necessity to design different IQA networks for ultrasound images with and without lesions. Due to the accuracy improvement for the images with lesions, our proposed global–local integrated IQA framework had better performance than the global BCNN network only, with PLCC 0.8851, SRCC 0.8834, Accuracy 0.8288, and RMSE 0.4541, which showed the advantage of our proposed global–local integrated framework.

**Figure 8:**
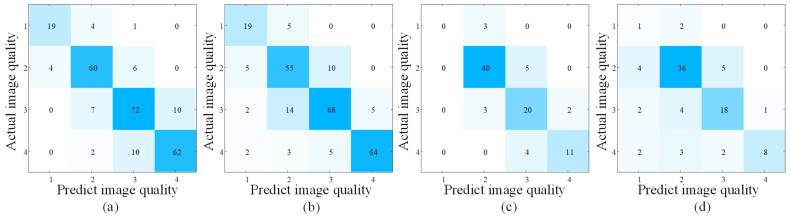
Confusion matrix of IQA experiments. (**a**) Result of global–local integrated framework for mixed data. (**b**) Result of BCNN method for mixed data. (**c**) Result of SR-IQA method for positive data. (**d**) Result of BCNN method for positive data. The colors on the diagonal of (**a**) is the darkest, which indicates that the method performs best.

Figure 8 shows the confusion matrix of IQA experiments. It can be noted that for the global BCNN network only, the image quality of some ultrasound images with lesions were incorrectly scored, reducing the performance of the overall IQA task. The reason can be explained in the two aspects. Firstly, the lesions produced hypoechoic dark areas in the ultrasound image, which may have affected the assessment of the global quality evaluation. Secondly, some images containing lesions had poor global quality, but the lesion areas were clear enough to help doctors in making their diagnosis; the doctor would then classify them as high-quality images. Therefore, implementing image quality assessment separately according to the existence of lesions significantly improved the consistency of the evaluation method with the physician’s clinical judgement.

### 3.8. Real-Time Performance

In practice, ultrasound image quality evaluation methods can be used to evaluate images acquired by physicians, to provide guidance to new physicians, or as image feedback for ultrasound autonomous scanning robots. Therefore, we should consider its Real-Time Performance. We have conducted real-time tests with the trained network to evaluate the quality of 257 ultrasound images from the test dataset. The experimental results show that the total time cost is 55.51 s, and the average time to evaluate the quality of a single image is 0.216 s. This is acceptable for a quality evaluation application. Of course, this is also relevant to the equipment on which the application is deployed.

## 4. Discussion and Conclusions

This paper presented a novel breast US image quality assessment framework integrating global IQA for ultrasound images without lesions and local SF-IQA for ultrasound images with lesions. The proposed SF-IQA used the lesion segmentation mask as the reference and transformed the medical ultrasound image assessment problem from no reference to reduced reference. To assess the image quality of the local lesion area, a comprehensive evaluation index, including the pixel-level features MSE and PSNR and the semantic-level features CR and SSIM, is proposed. Thanks to the specially designed image features and classification network for the lesion area, our proposed SF-IQA achieved a large improvement for quality assessment of ultrasound images with lesions (PLCC 0.8418 vs. 0.6606), proving the necessity of designing different IQA networks for ultrasound images with and without lesions. Due to the accuracy improvement for the images with lesions, our proposed global–local integrated IQA framework had better performance in the IQA task than the global BCNN network that did not consider the local lesion features, with PLCC improving from 0.8306 to 0.8851. The soft-reference method we propose can be extended to images containing cystic lesions, and other medical images where a clear visualization of key anatomical structures is required. For example, the area around the carotid artery in a carotid ultrasound image, or the cartilage and surrounding area in a knee ultrasound image. However, The IQA framework proposed in this research can still be further improved. Infiltrating cancer has unclear boundaries and may be judged as low quality by the IQA network. In our future work, benign–malignant decisions will be added to the IQA framework, along with the development of ultrasound image IQA methods for malignant lesions.

## Figures and Tables

**Table 1:** The numbers of breast ultrasound images with different quality scores.

Lesion	Samples				
	Score	1	2	3	4
Negative	Image	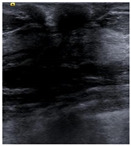	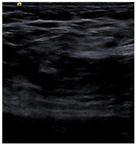	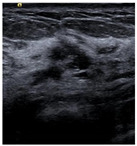	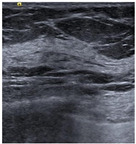
	Number	98	112	345	289
Positive	Image	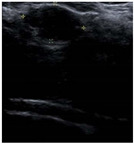	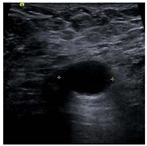	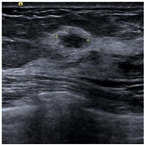	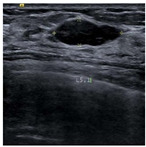
	Number	47	49	161	184

**Table 2:** Result of classification experiment.

Model	Accuracy of Validation Set	Accuracy of Test Set	Best Validation Epoch
ResNet18 without pre-training	0.966	0.953	15
ResNet18 with pre-training	0.970	0.965	11
CBAM-ResNet18 with pre-training	**0.989**	**0.992**	**5**

**Table 3:** Result of global IQA experiment.

Dataset	PLCC	SRCC	Accuracy	RMSE
Negative (A.3)	0.8964	0.8765	0.8402	0.4615

**Table 4:** Result of segmentation experiment.

Model	Pretrained	Val-Dice	Test-Dice
VGG16	ImageNet	0.8346 ± 0.0839	0.8633 ± 0.0936
PSPNet	ImageNet	0.8700 ± 0.1174	0.8789 ± 0.1024
DeepLab V3+	ImageNet	0.8968 ± 0.0560	0.8824 ± 0.0865
U-Res	ImageNet	0.9056 ± 0.1676	0.8932 ± 1.1895
ViT	ImageNet	0.9162 ± 0.0964	0.9065 ± 0.6352
U-Res-CBAM	ImageNet	**0.9272 ± 0.1155**	**0.9126 ± 0.0971**

**Table 5:** Result of local SF-IQA experiment.

Model	ValPLCC	ValSRCC	ValAccuracy	ValRMSE	TestPLCC	TestSRCC	TestAccuracy	TestRMSE
Vgg16	0.5587	0.5219	0.6232	0.7161	0.6381	0.6011	0.6250	0.6657
PSPNet	0.6546	0.6307	0.6289	0.6028	0.6830	0.7055	0.6364	0.6307
Deeplab v3+	0.6500	0.6516	0.6601	0.6584	0.7254	0.7118	0.6932	0.5839
U-Res	0.7735	0.7800	0.7507	0.5160	0.8031	0.7945	0.7500	0.5000
U-Res-CBAM	**0.8307**	**0.8430**	**0.8130**	**0.4516**	**0.8418**	**0.8462**	**0.8068**	**0.4395**
Ground-truth	0.9255	0.9394	0.9065	0.3058	0.8996	0.9251	0.8636	0.3693

**Table 6:** Result of global–local integrated IQA and global only IQA.

Dataset	Model	PLCC	SRCC	Accuracy	RMSE	95% CI of Acc
Positive (B.3)	SR-IQA	0.8418	0.8462	0.8068	0.4395	(0.7243, 0.8893)
Positive (B.3)	BCNN	0.6606	0.7062	0.7159	0.6946	(0.6217, 0.8101)
Mixed (A.3 + B.3)	Global-local	**0.8851**	**0.8834**	**0.8288**	**0.4541**	(0.7501, 0.9075)
Mixed (A.3 + B.3)	BCNN	0.8306	0.8338	0.8016	0.5649	(0.7183, 0.8849)

## Data Availability

The datasets presented in this article are not readily available because the hospital’s research ethics board does not permit sharing of clinical images.
